# Preoperative Albumin Infusion Reduced Pulmonary Complications in Elderly Patients With Hypoalbuminemia Undergoing Cardiac Surgery: A Single‐Center, Randomized, Double‐Blind, Controlled Pilot Trial

**DOI:** 10.1002/mco2.70840

**Published:** 2026-07-05

**Authors:** Jie Liu, Shiqiang Chen, Yunxiao Bai, Yong Lv, Yanting Wang, Zhenzhen Xu, Nianguo Dong, Qingping Wu

**Affiliations:** ^1^ Department of Anesthesiology The Second Affiliated Hospital Chongqing Medical University Chongqing China; ^2^ Department of Anesthesiology Union Hospital Tongji Medical College Huazhong University of Science and Technology Wuhan China; ^3^ Department of Cardiovascular Surgery Union Hospital Tongji Medical College Huazhong University of Science and Technology Wuhan China

**Keywords:** albumin, cardiopulmonary bypass, elderly patients, inflammatory cells, postoperative pulmonary complications

## Abstract

Hypoalbuminemia is a recognized risk factor for postoperative pulmonary complications (PPCs), but whether preoperative albumin infusion improves pulmonary outcomes after cardiac surgery remains unclear. In this single‐center, randomized, double‐blind, controlled pilot trial (ChiCTR2300076609), 80 elderly patients with preoperative albumin concentrations <40 g/L who underwent elective cardiac surgery with cardiopulmonary bypass were randomly assigned to receive 100 mL of 20% albumin (40 patients) or saline before surgery (40 patients). The primary outcomes were PPC severity and incidence during hospitalization, and perioperative immune cell changes were also explored. All randomized participants completed the trial. Compared with the controls, the patients in the albumin group had lower PPC severity (median [IQR], 1 [1–2] vs. 2 [2–3]; mean difference, −0.75; 95% CI, −1.15 to −0.35; *p* = 0.001) and a lower incidence of PPCs (30% vs. 57.5%; risk difference, −0.275; 95% CI, −0.48 to −0.07; *p* = 0.013). Additionally, the Th17 cell proportion and γδTreg cell proportion were significantly decreased and increased, respectively, in the albumin group after surgery (*p* < 0.001). These findings suggest that preoperative albumin supplementation may reduce PPC risk and modulate immune responses, warranting confirmation in larger multicenter trials.

## Graphical abstract

Elderly patients with preoperative hypoalbuminemia (< 40 g/L) undergoing cardiac surgery with cardiopulmonary bypass were randomized to receive 100 mL of 20% albumin or saline before surgery. Albumin infusion reduced the severity and incidence of PPCs and modulated immune balance. Patients who received albumin showed lower Th17 cell proportions and higher γδTreg cells at the end of surgery, suggesting improved perioperative immune homeostasis and reduced pulmonary risk.

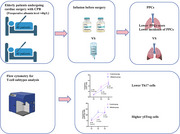

## 1 Introduction

Postoperative pulmonary complications (PPCs) are among the most common complications following cardiac surgery [1, 2], with an incidence reaching 50% [1]. PPCs not only prolong hospital and intensive care unit (ICU) stays but also significantly increase healthcare costs and mortality rates, imposing a substantial burden on healthcare systems [3]. Even mild PPCs have been associated with prolonged hospitalization and increased mortality [4]. Therefore, PPCs represent a major contributor to postoperative morbidity and mortality and remain a significant challenge in perioperative management.

Nearly all cardiac surgery patients experience some degree of pulmonary dysfunction [2, 5]. Existing studies have identified advanced age as an independent risk factor for PPCs [6], possibly because of age‐related declines in pulmonary function and immune competence in elderly patients. Other key risk factors include prolonged cardiopulmonary bypass (CPB) duration and hypoalbuminemia [7, 8]. In addition, intraoperative ventilation–perfusion mismatch and thoracic compression may contribute to alveolar collapse and increase the risk of atelectasis [9]. Therefore, targeting modifiable risk factors is essential for reducing the risk of PPCs in elderly cardiac surgery patients.

Hypoalbuminemia compromises the integrity of the protective endothelial glycocalyx, leading to the extravasation of albumin and fluid into the interstitial tissue, thereby impairing normal tissue perfusion. As it is a negative acute‐phase protein, serum albumin levels decrease during inflammatory states, directly influencing surgical outcomes [10]. Albumin exerts anti‐inflammatory effects through multiple mechanisms, including the regulation of inflammatory cell activity and transcription factor expression [11].

However, definitive evidence supporting the effectiveness of albumin infusion in reducing the risk of PPCs before cardiac surgery is lacking. While a precise threshold for serum albumin deficiency has not been established, in elderly patients, this threshold is likely approximately 40 g/L [8]. A randomized trial in off‐pump cardiac surgery patients revealed that preoperative infusion of 20% albumin significantly reduced the risk of acute kidney injury [12]. Additionally, our previous retrospective study of 660 CPB patients revealed that a serum albumin concentration < 40 g/L was an independent risk factor for PPCs [13].

To explore whether albumin can reduce the severity and incidence of PPCs in elderly patients undergoing cardiac surgery with CPB, we designed this study to evaluate the effects of exogenous albumin administration compared with 0.9% sodium chloride infusion. This study focuses specifically on elderly patients whose preoperative serum albumin concentration was less than 40 g/L, aiming to provide critical insights into the role of albumin infusion in reducing PPCs under these conditions.

## 2 Results

### 2.1 Patient Characteristics

**FIGURE 1:**
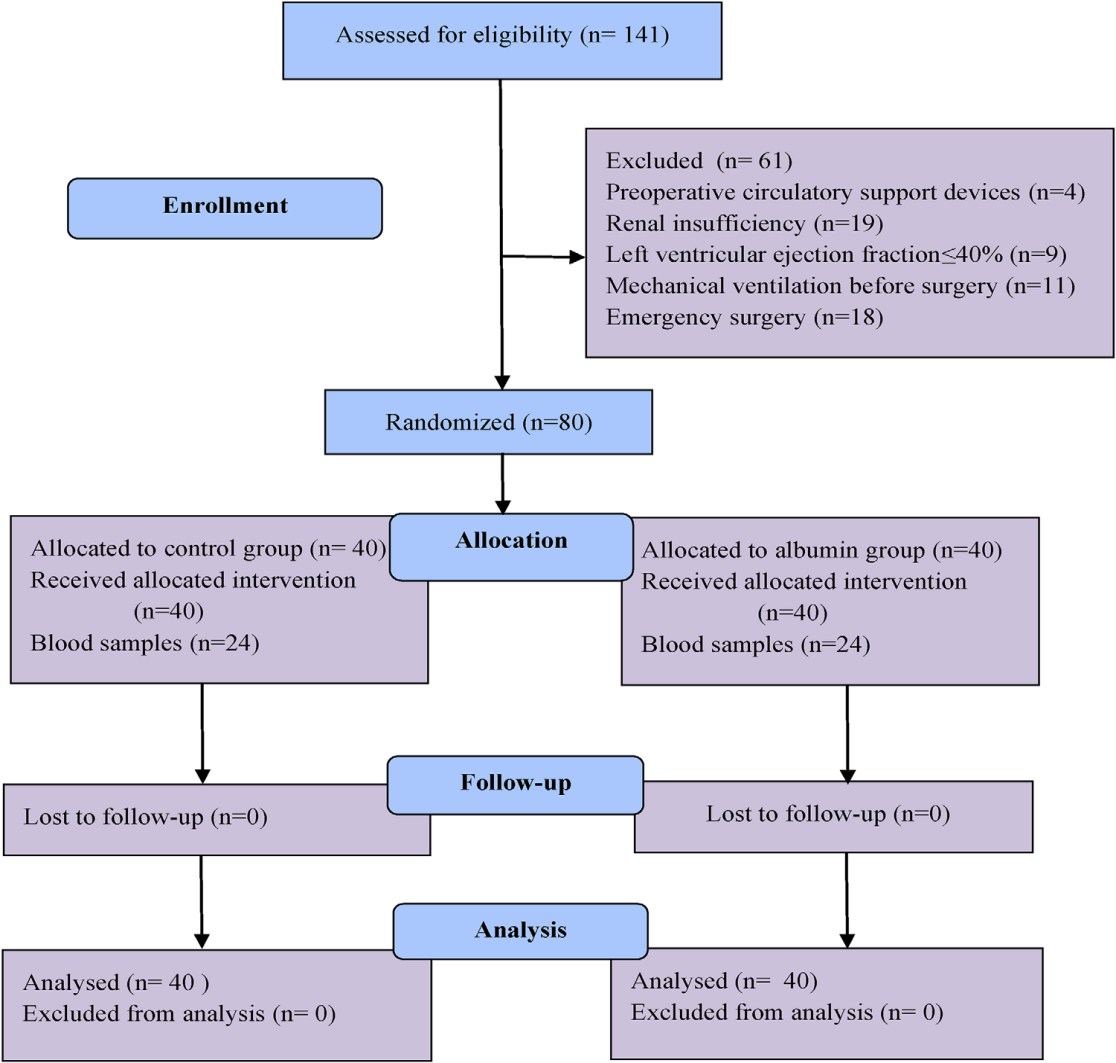
The flowchart of the trial.

A total of 141 eligible patients were screened from October 2023 to June 2024. After applying the exclusion criteria, 61 patients were excluded. Ultimately, 80 patients were enrolled and randomized into two groups: 40 patients were assigned to the albumin group and 40 patients were assigned to the control group. All randomized patients completed the trial protocol and were included in the final analysis (Figure 1). Table 1 presents the baseline characteristics, intraoperative features, and laboratory test results. The median preoperative albumin concentration was 37.15 g/L (36.08–37.8) in the control group and 36.65 g/L (35.72–38.6) in the albumin group, with no significant difference between the two groups (*p* = 0.927). Intraoperative albumin infusion was not administered in either group, and no significant differences were observed between the two groups for any variables.

**TABLE 1:** Baseline characteristics of all patients.

Variables	Control group (*n* = 40)	Albumin group (*n* = 40)	*p* value
Age, years	68 (66, 72)	68 (66, 71)	0.702
Male	27 (67.5)	24 (60)	0.485
BMI, kg/m^2^	23.03 ± 2.84	23.95 ± 3.08	0.165
Smoking	22 (55)	21 (52.5)	0.823
Alcohol	21 (52.5)	19 (47.5)	0.655
ASA IV	20 (50)	20 (50)	1
Hypertension	20 (50)	22 (55)	0.654
Diabetes	7 (17.5)	7 (17.5)	1
Coronary heart disease	21 (52.5)	22 (55)	0.823
Atrial fibrillation	11 (27.5)	10 (25)	0.799
COPD	5 (12.5)	9 (22.5)	0.239
Stroke	4 (10)	6 (15)	0.499
NYHA	1 (1,2)	2 (1,2)	0.328
LVEF, %	60 (59.75, 65)	61 (58.75, 65)	0.996
SpO_2_, %	98 (96.8, 99)	98 (97, 98)	0.41
EuroSCORE	4 (3, 5.25)	4 (3, 5)	0.5
ARISCAT score	50 (50, 50)	50 (50, 50)	0.638
Hemoglobin, g/L	122.42 ± 12.83	125 ± 13.63	0.387
ALT, U/L	19 (13.75, 29.25)	20 (16, 36)	0.223
AST, U/L	23 (20, 33)	24 (21.75, 32.25)	0.421
Albumin, g/L	37.15 (36.08, 37.8)	36.65 (35.72, 38.6)	0.927
Blood urea nitrogen, mmol/L	6.14 (5.38, 7.2)	6.18 (5.26, 7.67)	0.954
Creatinine, µmol/L	77.54 ± 18.84	77.56 ± 20.44	0.997
Type of surgery			0.834
CABG	12 (30)	10 (25)	
Valve	18 (45)	18 (45)	
Others	10 (25)	12 (30)	
Duration of surgery, min	265.18 ± 64.28	257.88 ± 64.03	0.612
Duration of anesthesia, min	332.65 ± 80.29	323.25 ± 66.26	0.57
CPB, min	114.97 ± 35.79	118.53 ± 31.74	0.64
Aortic occlusion time, min	72.92 ± 27.47	79.6 ± 26.29	0.27
Intraoperative crystalloid, mL	1500 (1000, 1500)	1500 (1000, 2000)	0.295
Intraoperative colloid, mL	0 (0, 50)	0 (0, 0)	0.341
RBC transfusion, U	1 (0, 2)	0.25 (0, 1.62)	0.32
Plasma transfusion, mL	0 (0, 150)	0 (0, 150)	0.634
Pre‐filled crystal, mL	500 (500, 600)	500 (500, 500)	0.282
Pre‐filled with colloid, mL	800 (500, 800)	750 (500, 800)	0.36
Urine output, mL	1500 (1142.5, 2075)	1630 (1100, 1910)	0.624

*Note*: Data are presented as *n* (%), mean (standard deviation), or median (interquartile range).

Abbreviations: ALT, alanine aminotransferase; ARISCAT, the assess respiratory risk in surgical patients in Catalonia; ASA, American society of anesthesiologists; AST, aspartate aminotransferase; BMI, body mass index; CABG, coronary artery bypass graft; COPD, chronic obstructive pulmonary disease; CPB, cardiopulmonary bypass; EuroSCORE, the European system for cardiac operative risk evaluation; LVEF, left ventricular ejection fraction; NYHA, New York Heart Association; RBC, red blood cells; SPO2, peripheral oxygen saturation.

Compared to preoperative albumin levels, the reduction in albumin concentration on postoperative day 1 was attenuated in the albumin group compared to the control group, with a mean difference (MD) of 0.83 g/L (95% confidence interval [CI]: 0.12–1.55, *p* = 0.034). However, by postoperative Day 7, no significant difference in albumin concentration changes was observed between the two groups (MD: 0.8 g/L, 95% CI: −1.08 to 2.67, *p* = 0.343; Figure S1).

### 2.2 Primary Outcomes

**FIGURE 2:**
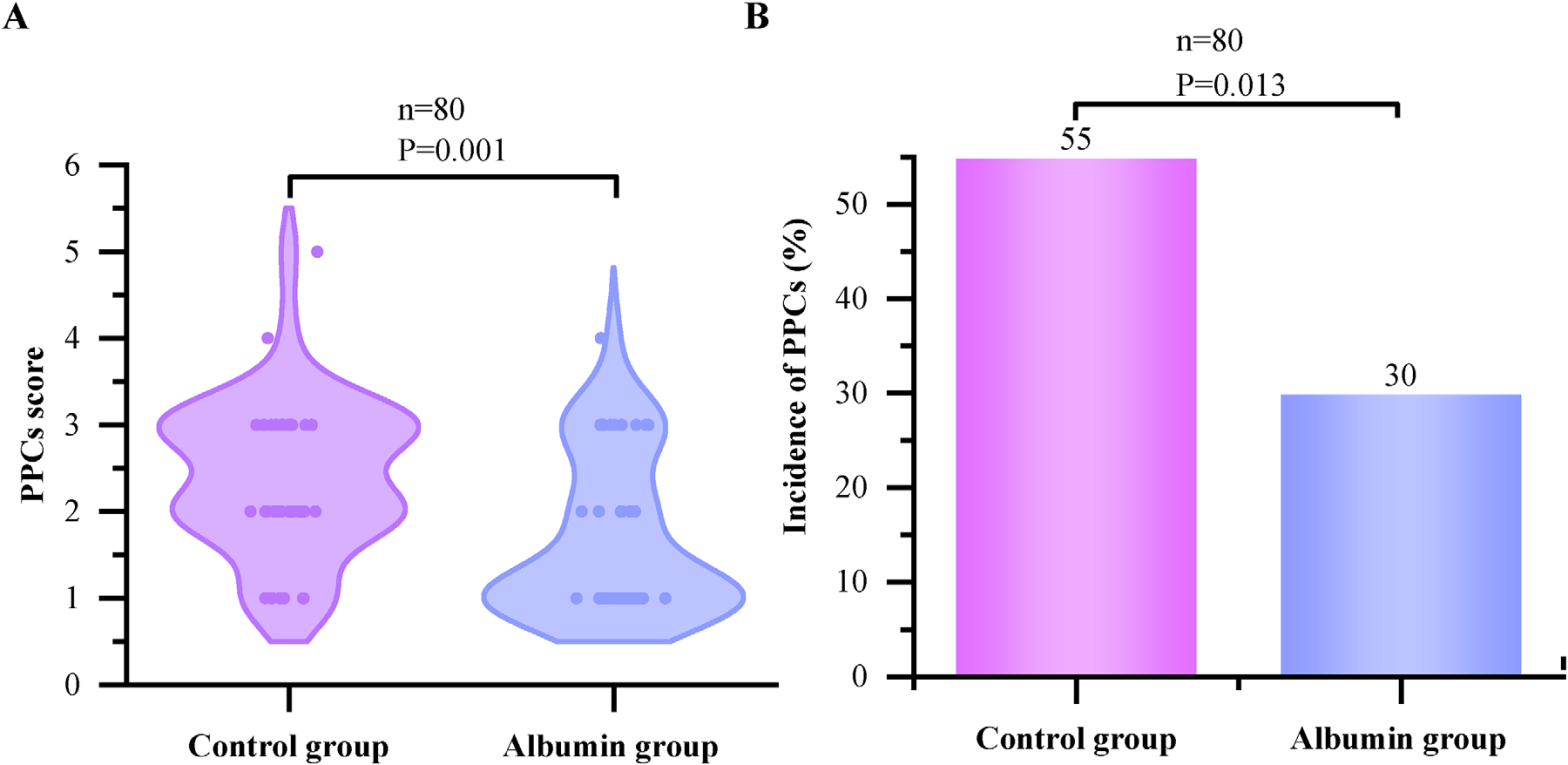
The scores and incidences of PPCs from the albumin group and the control group. (A) The scores of PPCs in the albumin and control groups. (B) The incidences of PPCs in the albumin and control groups. PPCs, postoperative pulmonary complications.

The pulmonary complication scores were 1 (1–2) in the albumin group and 2 (2–3) in the control group, with the albumin group demonstrating lower scores (MD: −0.75; 95% confidence interval [CI]: −1.15 to −0.35; *p* = 0.001) (Figure 2A). The specific distribution of PPC scores for both groups is shown in Figure S2, with the albumin group having a higher concentration of lower scores.

Furthermore, the incidence of pulmonary complications was 30% in the albumin group compared with 57.5% in the control group, indicating a significant difference (risk difference [RD]: −0.275; 95% CI: −0.48 to −0.07; *p* = 0.013; Figure 2B).

We conducted a detailed analysis of specific pulmonary complications in patients (Table 2). The incidence of pneumonia was lower in the albumin group (15%) than in the control group (45%) (RD: −0.3; 95% CI: −0.49 to −0.11; *p* = 0.003), and the incidence of atelectasis was also lower in the albumin group (10%) than in the control group (32.5%) (RD: −0.225; 95% CI: −0.40 to −0.05; *p* = 0.029). In the albumin group, the incidences of thoracentesis, acute respiratory distress syndrome (ARDS), pneumothorax, reintubation, and postoperative mechanical ventilation lasting more than 48 h were 10%, 7.5%, 5%, 0% and 2.5%, respectively. In contrast, the corresponding incidences in the control group were 15% (*p* = 0.499), 15% (*p* = 0.479), 2.5% (*p* = 1), 2.5% (*p* = 1), and 5% (*p* = 1), respectively.

**TABLE 2:** Specific postoperative pulmonary complications.

Variables	Control group (*n* = 40)	Albumin group (*n* = 40)	*p* value
Pneumonia	18 (45)	6 (15)	0.003
Atelectasis	13 (32.5)	4 (10)	0.029
Pleural effusion	6 (15)	4 (10)	0.499
ARDS	6 (15)	3 (7.5)	0.479
Pneumothorax	1 (2.5)	2 (5)	1
Re‐intubation	1 (2.5)	0 (0)	1
Postoperative mechanical ventilation duration exceeding 48 h	2 (5)	1 (2.5)	1

*Note*: Data are presented as *n* (%).

Abbreviation: ARDS, acute respiratory distress syndrome.

### 2.3 Secondary Outcomes

The postoperative hospital stay and ICU length of stay were shorter in the albumin group than in the control group (hospital stay: median 10 days [5.75–13] vs. 11 days [9–14]; MD: −2.22; 95% CI: −4.36 to −0.09; *p* = 0.049; ICU stay: 43.5 h [37.75–67] vs. 66 h [44–84.25]; MD: −20.63; 95% CI: −45.03 to −3.78; *p* = 0.015). There was no significant difference in the duration of postoperative mechanical ventilation between the two groups (*p* = 0.718). The incidence of acute kidney injury and all‐cause mortality did not differ significantly between the albumin group and the control group (*p* = 0.39 and *p* = 1, respectively) (Table 3).

**TABLE 3:** Secondary outcome.

Variables	Control group (*n* = 40)	Albumin group (*n* = 40)	*p* value
Postoperative length of hospital stay, days	11 (9, 14)	10 (5.75, 13)	0.049
AKI	6 (15)	9 (22.5)	0.39
All‐cause mortality	1 (2.5)	0 (0)	1
Postoperative mechanical ventilation duration, h	18 (10.75, 20.5)	17 (10.75, 20.25)	0.718
ICU length of stay, h	66 (44, 84.25)	43.5 (37.75, 67)	0.015
Cardiovascular complications	5 (12.5)	6 (15)	0.745

*Note*: Data are presented as *n* (%) or median (interquartile range).

Abbreviations: AKI, acute kidney injury; ICU, intensive care unit.

### 2.4 Subgroup Analysis

Subgroup analyses demonstrated that compared with the control group, the albumin group consistently had lower PPC scores across most subgroups (Table S1). Among patients aged < 70 years and ≥ 70 years, the PPC scores were significantly lower in the albumin group (both *p* < 0.05). Similar findings were observed in both female and male patients, with significantly reduced PPC scores in the albumin group (*p* = 0.014 and *p* = 0.018, respectively). Stratified by preoperative albumin concentration, patients with levels < 35 g/L and 35–40 g/L showed significantly lower PPC scores in the albumin group than in the control group (*p* = 0.043 and *p* = 0.007, respectively). By surgery type, patients undergoing CABG or valve surgery showed significantly lower PPC scores in the albumin group than in the control group (*p* = 0.023 and *p* = 0.042, respectively), with no significant difference for other surgeries (*p* = 0.106).

### 2.5 Immunological Changes

**FIGURE 3:**
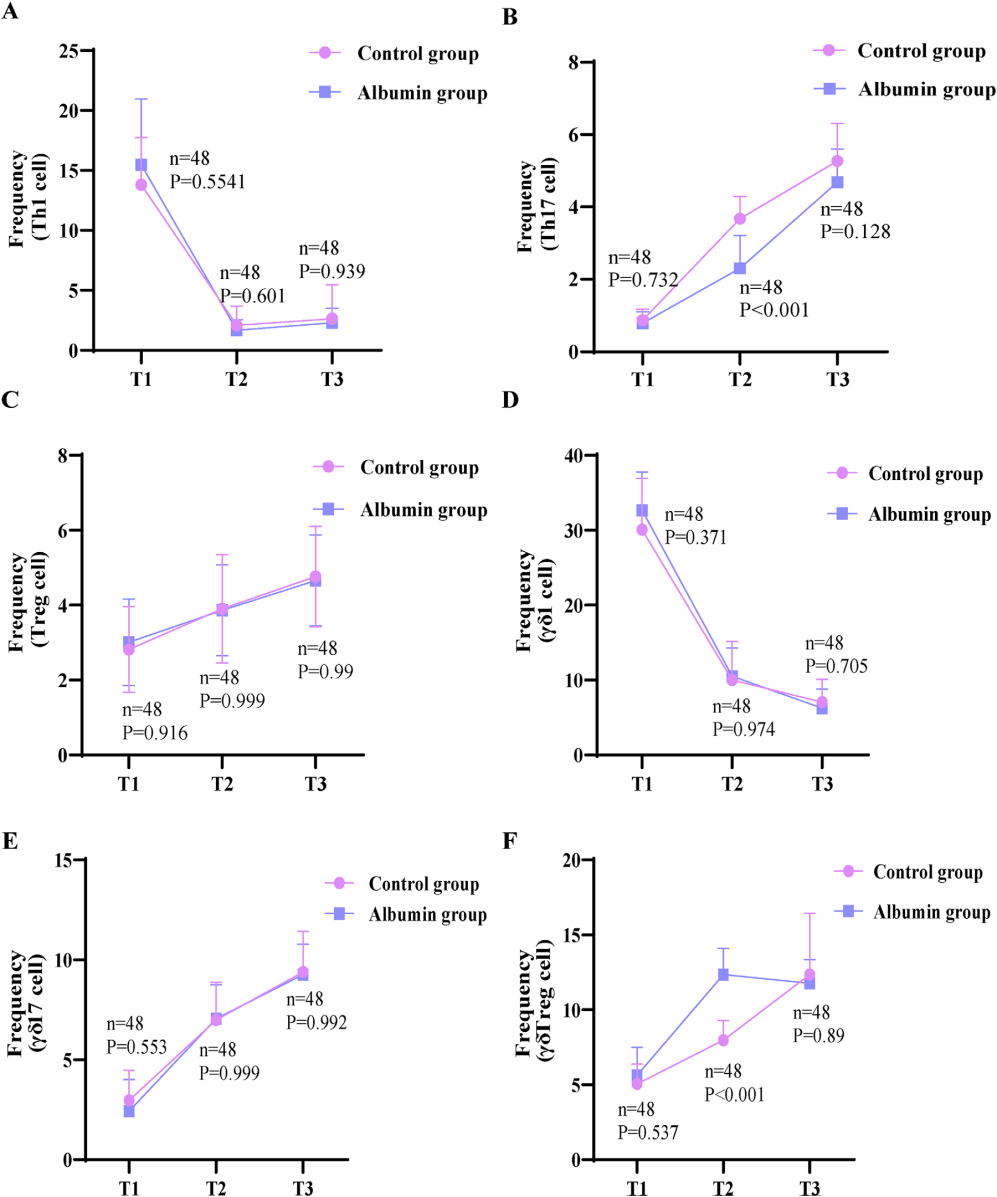
Changes in T‐cell subsets preoperatively, at the end of surgery, and 24 h postoperatively. (A) Changes in T helper1 cell subsets during the perioperative period. (B) Changes in Th17 cell subsets during the perioperative period. (C) Changes in Treg cell subsets during the perioperative period. (D) Changes in γδ1 cell subsets during the perioperative period. (E) Changes in γδ17 cell subsets during the perioperative period. (F) Changes in γδTreg cell subsets during the perioperative period. Data are presented as mean ± standard deviation. Comparisons were analyzed using two‐way repeated‐measures analysis of variance, followed by Bonferroni‐adjusted post hoc tests. T1, preoperative; T2, end of surgery; T3, 24 h postoperatively; T helper, Th; regulatory T, Treg.

After informed consent was obtained from 48 patients (24 in the albumin group and 24 in the control group), blood samples were collected before surgery, immediately after surgery, and 24 h after surgery for flow cytometry analysis. The baseline characteristics of patients whose blood samples were collected were not significantly different between the two groups (Table S2). The results of T cell and γδT cell subsets are shown in Figure 3. The proportion of T helper (Th) 1 cells was high before surgery, but there were no significant differences between the two groups immediately after surgery or 24 h after surgery. In contrast, the proportion of Th17 cells, which was initially low before surgery, increased after surgery, with lower levels in the albumin group than in the control group immediately after surgery (*p* < 0.001). The regulatory T (Treg) cell proportion did not significantly differ between the two groups at any time point.

With respect to the γδT cell subsets, the preoperative γδTh1 cell proportion was high, but no significant differences were observed between the two groups immediately after surgery or 24 h after surgery. While the proportion of γδ17 cells tended to increase after surgery, the difference was not significant between the two groups. Notably, the γδ Treg cell proportion was greater in the albumin group than in the control group immediately after surgery (*p* < 0.001). The level of interleukin‐10 (IL‐10) expressed by γδTreg cells in the albumin group was greater than that in the control group immediately after surgery (*p* = 0.002). Furthermore, no significant differences were observed between the two groups in terms of intracellular IL‐10 levels expressed by Treg cells or intracellular transforming growth factor‐β (TGF‐β) levels expressed by both Treg and γδTreg cells immediately after surgery or at 24 hours after surgery (Figure S3).

Spearman correlation coefficients between changes in various T‐cell proportions (relative to baseline) and PPC scores are presented in Table S3. Changes in the proportion of Th17 cells after surgery (post‐surgery minus baseline) were positively correlated with PPC scores (correlation coefficient = 0.312; *p* = 0.031), whereas changes in the proportion of γδTreg cells after surgery (post‐surgery minus baseline) were negatively correlated with PPC scores (correlation coefficient = −0.302; *p* = 0.037).

## 3 Discussion

Among elderly patients who underwent CPB, preoperative albumin administration reduced the PPC score (*p* = 0.001) and led to a 27.5% reduction in the overall incidence of PPCs (*p* = 0.013). Notably, compared with the control group, the albumin group had lower incidences of postoperative infections and pulmonary atelectasis. Additionally, the proportion of Th17 cells markedly decreased in the albumin group, which was accompanied by an increase in the proportion of γδTreg cells after surgery. These findings suggest that preoperative albumin administration may contribute to immune modulation, potentially enhancing immune tolerance and reducing PPCs.

Albumin, the most abundant circulating protein in the blood, is crucial for maintaining normal physiological functions. Retrospective studies have linked decreased preoperative albumin levels to an elevated risk of PPCs. However, the clinical benefits of albumin supplementation remain debated. A meta‐analysis of 20 studies with 22,553 cardiac surgery patients revealed a significant association between hypoalbuminemia and increased long‐term mortality [14]. In contrast, another meta‐analysis of 42 studies on intravenous albumin administration in cardiovascular surgeries revealed no reduction in morbidity or mortality [15]. Most research on albumin transfusion focuses on critically ill patients and its role in correcting hypoalbuminemia, particularly in cardiac surgery for CPB initiation and volume replacement [16, 17]. However, there is limited evidence on the impact of albumin supplementation on PPCs in elderly patients with hypoalbuminemia who underwent CPB.

Hypoalbuminemia is widely recognized as a critical preoperative risk factor and is strongly associated with prolonged mechanical ventilation, extended hospital stays, and increased incidences of acute kidney injury, infections, and mortality [18, 19]. As it is a negative acute‐phase protein, changes in albumin levels primarily reflect inflammatory responses and disease severity in acute conditions [20, 21].

A prospective study involving 2465 emergency department patients revealed that elevated inflammatory marker levels and nutritional risk were independently associated with hypoalbuminemia, with low serum albumin levels, increased C‐reactive protein levels, and heightened nutritional risk identified as independent predictors of mortality [22]. In patients with acute lung injury, albumin therapy has been shown to improve plasma thiol‐dependent antioxidant status and reduce the degree of oxidative protein damage [23]. Animal studies further support the protective role of albumin infusion in lung injury, with a mouse model demonstrating that infusion of 25% albumin significantly inhibited lipid peroxidation and alleviated lung injury by modulating neutrophil activity [24]. Similarly, our study revealed that administering 20% albumin to patients whose preoperative albumin levels were below 40 g/L effectively reduced the severity and incidence of PPCs.

In our study, albumin administration significantly reduced the incidence rates of postoperative pneumonia and atelectasis. Several physiological mechanisms may contribute to these findings. Albumin increases plasma oncotic pressure, thereby reducing pulmonary interstitial edema and improving lung compliance [25]. In addition, albumin possesses anti‐inflammatory and endothelial protective properties that may decrease capillary permeability and limit pulmonary exudation [26], thereby exerting protective effects on the lung during the perioperative period. Notably, atelectasis itself is closely associated with inflammatory responses [27]. Therefore, albumin supplementation may reduce the risks of postoperative pneumonia and atelectasis by alleviating pulmonary edema, improving lung function, and modulating inflammatory responses.

Given that CD4^+^ T cells and γδ T cells represent key functional subsets of CD3^+^ T cells with important roles in immune regulation, we focused on examining their subpopulations, including Th1, Th17, Treg, γδ1, γδ17, and γδTreg cells. Perioperative immune alterations were analyzed using repeated‐measures analysis of variance (ANOVA) to account for within‐subject temporal variation across baseline, end‐of‐surgery, and 24‐h post‐operative time points, rather than relying solely on cross‐sectional comparisons. Although patients in the albumin group exhibited relatively lower Th17 proportions and higher γδTreg proportions at the end of surgery, postoperative changes in Th17 cell proportions were positively correlated with PPC scores, whereas changes in γδTreg cell proportions were negatively correlated with PPC scores. Taken together, these findings should be interpreted with caution. During CPB, hemodilution, intraoperative blood loss, and perioperative fluid management may alter circulating immune cell proportions, thereby influencing the proportions detected by flow cytometry. Given that relative cell proportions rather than absolute counts were measured in this study, the potential impact of hemodilution and fluid redistribution cannot be fully excluded. In addition, high‐concentration albumin infusion may affect intravascular colloid osmotic pressure, thereby influencing the redistribution of immune cells between the intravascular and extravascular compartments, particularly for rare subsets such as γδTreg cells. Therefore, the observed postoperative changes in immune cell proportions may reflect both true immunomodulatory effects and, in part, redistribution effects driven by perioperative fluid dynamics. PPCs following cardiac surgery are closely associated with systemic inflammatory responses [28, 29]. From a mechanistic perspective, Th17 cells are key pro‐inflammatory cells that secrete cytokines such as IL‐17A, promote neutrophil recruitment, and induce the release of inflammatory mediators including tumor necrosis factor‐alpha and IL‐6, thereby exacerbating inflammation [30, 31]. Previous studies have also shown that Th17 cells and IL‐17 expression levels are significantly elevated in patients with ARDS, contributing to worsening inflammation [32, 33, 34]. In contrast, γδTreg cells play critical immunosuppressive roles in conditions such as infection control, tumor immunity, and graft‐versus‐host disease, primarily through the secretion of IL‐10 and TGF‐β [35, 36, 37]. Characterized by the coexpression of γδT‐cell receptor and forkhead box P3, γδTreg cells have been shown to suppress immune responses in various disease models by inhibiting the activation and function of antigen‐specific T cells, thereby reducing pathological immune reactions [38, 39]. Additionally, γδTregs have been shown to have anti‐inflammatory effects in diseases such as asthma [40].

In the present study, preoperative albumin infusion induced changes in the proportions of Th17 and γδTreg cells, which may suggest a potential immunomodulatory signal. To further evaluate the clinical relevance of these immunological alterations, we performed Spearman correlation analyses. The results showed that changes in Th17 cells at the end of surgery were positively correlated with PPC scores, whereas changes in γδTreg cells were also significantly associated with PPC scores. These findings suggest that perioperative immune modulation, reflected by alterations in pro‐inflammatory and Treg cell subsets, may be linked to the severity of PPCs. Given the central role of inflammation in PPC development, we further explored the potential mechanistic implications. Inflammatory processes can reduce hepatic albumin synthesis and increase capillary permeability, leading to decreased serum albumin levels [41]. Albumin concentration is widely regarded as a marker of inflammatory burden and systemic stress rather than a purely nutritional indicator [22, 33]. In the perioperative setting, excessive inflammation is closely linked to the development of PPCs. Therefore, a relative attenuation of pro‐inflammatory responses (e.g., reduced Th17 activity), together with a more balanced regulatory immune profile, may reflect a more controlled inflammatory state, which may be associated with improved postoperative pulmonary outcomes.

However, the immunological endpoints in this study were exploratory in nature, and the sample size was relatively limited, preventing definitive mechanistic conclusions. Future studies with larger sample sizes, incorporating absolute immune cell counts and plasma volume‐adjusted analyses, are warranted to further validate the potential immunomodulatory effects of albumin infusion in the perioperative setting.

This study has several limitations. First, the relatively small sample size and single‐center design may limit the generalizability of the findings and may have led to an overestimation of the treatment effect. Future multicenter studies with larger sample sizes are warranted to further clarify the potential role of albumin infusion in reducing PPCs in patients with hypoalbuminemia. Second, this study relied on blood samples to indirectly assess systemic inflammatory responses, and local pulmonary inflammation was not directly evaluated. In future studies, bronchoalveolar lavage fluid should be collected to directly assess changes in pulmonary inflammatory cells, thereby providing a more comprehensive understanding of the mechanisms through which albumin alleviates PPCs in patients with hypoalbuminemia. Third, pulmonary function test data were not available. However, surrogate indicators, including chronic obstructive pulmonary disease incidence, preoperative SPO_2_ levels, and the ARISCAT score, were used to reflect preoperative pulmonary function status. Fourth, detailed intraoperative respiratory parameters were not recorded. However, mechanical ventilation was strictly standardized and uniformly applied across both groups, which is unlikely to have materially affected the comparability between groups. Finally, the incidence of PPCs in our cohort was relatively high; however, this reflects the inclusion of high‐risk elderly individuals who underwent CPB.

## 4 Conclusion

In the context of CPB surgery in elderly patients, preoperative albumin supplementation reduced both the severity and incidence of PPCs in patients with preoperative albumin concentrations less than 40 g/L. Additionally, a decrease in the proportion of Th17 cells and an increase in the proportion of γδTreg cells were observed, suggesting that albumin may exert its immunomodulatory effects by changing the proportions of crucial immune cell subsets, contributing to lung protection. These findings highlight the potential role of albumin in modulating the immune response and preventing PPCs, particularly in patients with hypoalbuminemia. However, large‐scale randomized clinical trials are needed to confirm these observations.

## 5 Materials and Methods

### 5.1 Patients

This double‐blind randomized clinical trial was conducted at a tertiary care teaching hospital in Wuhan Union Hospital between October 2023 and June 2024. The trial adhered strictly to the principles outlined in the Declaration of Helsinki and was registered in October 2023 (registration number: ChiCTR2300076609). Ethical approval was obtained from the Ethics Committee of Wuhan Union Hospital (Ethics approval number: 2023‐0472‐03). Written informed consent was obtained from all participants prior to enrollment. The study followed the Consolidated Standards of Reporting Trials guidelines. The full trial protocol can be found in the Supporting Information Materials.

Adult patients were consecutively enrolled on the basis of the following criteria: age ≥ 65 years; undergoing primary cardiac surgery under CPB, including coronary artery bypass grafting, valve repair or replacement, and aortic surgery; a preoperative serum albumin concentration between 30 and 40 g/L; and signed informed consent. The exclusion criteria were as follows: history of allergy to albumin; preoperative use of high‐dose positive inotropic agents, intra‐aortic balloon pumps, extracorporeal membrane oxygenation, or ventricular assist devices; preoperative renal insufficiency (serum creatinine ≥ 1.5 mg/dL); dialysis dependency; left ventricular ejection fraction ≤ 40%; mechanical ventilation within 7 days prior to surgery; and emergency procedures.

### 5.2 Randomization and Blinding

Patients who met the inclusion criteria were randomly assigned to either the albumin group or the saline group at a 1:1 ratio using a computer‐generated randomization sequence created by a statistician independent of the clinical study. Treatment assignments were made through sequentially numbered opaque sealed envelopes managed by the study coordinator and distributed in order. The trial was blinded to the participants. Follow‐up personnel and data analysts were also blinded.

The study medications included human albumin (Kilifor, Spain; 50 mL: 10 g) and placebo (saline). Both were stored under airtight conditions in individually wrapped boxes to ensure anonymity and maintain the trial's double‐blind design. Albumin was dispensed according to the patient's enrollment number and recorded on the case report form. Patients, researchers, follow‐up staff, and data analysts remained blinded to ensure the objectivity and reliability of the results.

### 5.3 Intervention

After patients entered the operating room and intravenous access was established, infusion of albumin (50 mL per dose, twice, for a total of 100 mL) or 100 mL of normal saline was initiated immediately. All patients received a standardized anesthesia protocol, and surgeries were performed by highly experienced surgical teams.

### 5.4 Anesthesia and Perioperative Management

All patients underwent a standardized anesthesia protocol to minimize variability in pulmonary outcomes. After radial artery puncture under local anesthesia, patients were intubated and mechanically ventilated using volume‐controlled ventilation. Anesthesia was maintained with propofol and remifentanil as primary agents, and a BIS index of 40–60 was maintained. After surgery, patients were transferred to the cardiac surgery ICU for further management. The attending physician tailored their perioperative care, including anesthesia and analgesia regimens, fluid management, transfusion strategies, and respiratory physiotherapy, to meet the individual needs of each patient.

Mechanical ventilation was performed using a lung‐protective strategy with volume‐controlled ventilation, a tidal volume of 6–8 mL/kg ideal body weight, and an inspiratory‐to‐expiratory ratio of 1:2. The respiratory rate was adjusted to maintain an end‐tidal carbon dioxide level of 35–45 mmHg. The inspired oxygen fraction was initially set at 100% during tracheal intubation, then reduced to approximately 60% after intubation, and subsequently titrated to maintain peripheral oxygen saturation (SPO_2_ ≥ 96%) during surgery. Positive end‐expiratory pressure of 3–5 cmH_2_O was applied as part of routine clinical practice. During CPB, mechanical ventilation was discontinued according to institutional routine practice and resumed after weaning from CPB. All patients received a standardized ventilation protocol throughout the procedure.

### 5.5 Outcomes

The primary outcomes of this study were the PPC scores and the incidence of PPCs during the hospital stay. Secondary outcomes included postoperative duration of invasive mechanical ventilation, incidence of acute kidney injury, cardiovascular complications, length of ICU stay, postoperative length of stay, and all‐cause mortality during the hospital stay.

All patients underwent bedside chest radiographs on postoperative Days 1, 3, and 5, followed by weekly imaging during hospitalization unless otherwise indicated by the attending clinician. All chest radiograph and follow‐up data were analyzed independently by two trained investigators, and both were blinded to patient grouping information. The final score for pulmonary complications was determined on the basis of an assessment of concordance between chest radiograph findings and clinical presentation. The PPC scores were based on the scoring scale published by Kroenke et al. [42] and ranged from 0 to 5 points. PPCs with a score ≥ 3 were recorded, along with the incidence of complications.

### 5.6 Blood Analysis

A subset of patients provided additional informed consent for blood sample collection and immunological analysis. Arterial blood samples were obtained from these patients before the administration of albumin or saline and 24 h after surgery for flow cytometry analysis of Th1 cells, Th17 cells, Treg cells, γδ1 cells, γδ17 cells, and γδTreg cells.

During the experiment, whole blood samples were first subjected to erythrocyte lysis to remove erythrocytes, followed by the collection of leukocytes, sequential cell stimulation, dead‐viable staining, surface staining, and intracellular staining. After staining was completed, the cells were washed twice with PBS and resuspended in PBS before data collection was performed using a FACS Fortessa flow cytometer (BD, San Diego, CA, USA). The antibodies used in the experiments included APC‐H7‐CD3, BV605‐CD4, BV421‐TCRγδ, APC‐IL‐17A, BV510‐IFN‐γ, PE‐Foxp3, PE‐CY7‐TGF‐β1, BB700‐IL‐10, and an isotype‐matched control IgG, which were purchased from BD PharMingen or BioLegend (San Diego, CA, USA). The collected flow cytometry data were analyzed by FlowJo software (BD, San Diego, CA, USA) to ensure the accuracy and consistency of the data.

### 5.7 Sample Size

On the basis of the published literature [42, 43] and the results of the preliminary test, we estimated that the difference in PPC scores between the intervention and control groups would be 0.3, with a standard deviation of 0.45. With respect to the incidence of PPCs, we assumed a rate of 60% in the control group and anticipated a 50% relative reduction in the intervention group. Using a two‐sided test with a significance level (α) of 0.05 and a power of 80%, the required sample size was calculated separately for each outcome. Sample size estimation based on PPC scores resulted in a requirement of 74 patients, whereas the calculation based on PPC incidence indicated a requirement of 58 patients. To ensure sufficient statistical power, the larger sample size of 74 was adopted. Accounting for a dropout rate of 5%, the final recruitment target was set at 80 patients.

### 5.8 Statistical Analysis

The baseline characteristics of the patients were summarized using descriptive statistics. Continuous variables with a normal distribution are presented as the mean ± standard deviation (mean ± SD), whereas nonnormally distributed continuous variables are expressed as the median and interquartile range (median [IQR]). Categorical variables are described as frequencies and percentages (*n*, %). Between‐group comparisons were conducted using the independent samples *t* test or the Mann–Whitney *U* test. Categorical variables were compared using the chi‐square test or Fisher's exact test, as appropriate. For repeated measures, ANOVA with the Bonferroni post hoc correction was used. Additionally, Spearman correlation analysis was performed to examine the association between changes in inflammatory cell counts and PPC scores. Subgroup analyses were performed according to preoperative subgroup analyses for the PPC severity score, the primary continuous outcome of the study. Patients were stratified according to preoperative serum albumin levels (< 35 g/L and 35–40 g/L) on the basis of the clinically relevant thresholds for hypoalbuminemia. Additional age, sex, and surgical type subgroup analyses were performed to evaluate the consistency of the treatment effect across different patient characteristics and procedures. A two‐sided *p* value of less than 0.05 was considered to indicate statistical significance. All the statistical analyses and visualizations were performed using SPSS software version 25.0 (IBM, Armonk, NY, USA), Origin (OriginLab Corporation, Northampton, MA, USA), and GraphPad Prism (GraphPad Software, San Diego, CA, USA).

## Author Contributions

**Jie Liu**: conceptualization, methodology, formal analysis, writing – original draft. **Shiqiang Chen**: software, formal analysis, visualization. **Yunxiao Bai**: validation, resources, data curation. **Yong Lv**: data curation, formal analysis. **Yanting Wang**: software, data curation, supervision, visualization. **Zhenzhen Xu**: methodology, validation, supervision, writing – review and editing. **Nianguo Dong**: resources, supervision, writing– review and editing. **Qingping Wu**: conceptualization, funding acquisition, resources, supervision, writing – review and editing. All authors have read and approved the final manuscript.

## Funding

This study was supported by the National Key Research and Development Program of China (Grant No. 2023YFC2506901), National Key Research and Development Program of China (Grant No. 2018YFC2001903), and the National Natural Science Foundation of China (Grant No. 81873952).

## Ethics Statement

The trial was conducted in strict accordance with the ethical principles outlined in the Declaration of Helsinki and was approved by the Ethics Committee of Wuhan Union Hospital (approval number: 2023‐0472‐03). Written informed consent was obtained from all participants prior to enrollment. The trial was registered at the Chinese Clinical Trial Registry (ChiCTR) (registration number: ChiCTR2300076609) on October 12, 2023.

## Conflicts of Interest

The authors declare no conflicts of interest.

## Supporting information




**Table S1**: Subgroup analyses of PPCs scores stratified by age, gender, preoperative albumin level, and surgical type.
**Table S2**: Baseline characteristics of patients with collected blood sampled.
**Table S2**: Spearman correlation analysis between T cell subtypes and PPCs scores.
**Figure S1**: Changes in albumin concentration on postoperative day 1 and day 7 relative to preoperative levels.
**Figure S2**: The PPCs score distribution in the albumin group and the control group. (A) The PPCs score distribution in the albumin group. (B) The scores of PPCs in the control group. PPCs, postoperative pulmonary complications.
**Figure S3**: Changes in intracellular factors within perioperative Treg cells and γδTreg subsets. (A) Changes in IL‐10 within Treg cells during the perioperative period. (B) Changes in TGF‐β within Treg cells during the perioperative period. (C) Changes in IL‐10 within γδTreg cells during the perioperative period. (D) Changes in TGF‐β within γδTreg during the perioperative period. Data are presented as mean ± standard deviation. Comparisons were analyzed using two‐way repeated‐measures analysis of variance, followed by Bonferroni‐adjusted post hoc tests. T1, preoperative; T2, end of surgery; T3, 24 hours postoperatively; regulatory T, Treg; Interleukin‐10, IL‐10; transforming growth factor beta, TGF‐β.

**Supporting Information**: mco270840‐sup‐0002‐SuppMat.docx

## Data Availability

The data that support the findings of this study are available from the corresponding author upon reasonable request.
