# Supplementary material for: Preoperative Albumin Infusion Reduced Pulmonary Complications in Elderly Patients With Hypoalbuminemia Undergoing Cardiac Surgery: A Single‐Center, Randomized, Double‐Blind, Controlled Pilot Trial
Source: MedComm (2020). 2026 Jul 5;7(7):e70840. doi: 10.1002/mco2.70840 (PMC13334133; doi:10.1002/mco2.70840)
Supplement: Supplementary file 1 — Table S1: Subgroup analyses of PPCs scores stratified by age, gender, preoperative albumin level, and surgical type. Table S2: Baseline characteristics of patients with collected blood sampled. Table S2: Spearman correlation analysis between T cell subtypes and PPCs scores. Figure S1: Changes in albumin concentration on postoperative day 1 and day 7 relative to preoperative levels. Figure S2: The PPCs score distribution in the albumin group and the control group. (A) The PPCs score distribution in the albumin group. (B) The scores of PPCs in the control group. PPCs, postoperative pulmonary complications. Figure S3: Changes in intracellular factors within perioperative Treg cells and γδTreg subsets. (A) Changes in IL‐10 within Treg cells during the perioperative period. (B) Changes in TGF‐β within Treg cells during the perioperative period. (C) Changes in IL‐10 within γδTreg cells during the perioperative period. (D) Changes in TGF‐β within γδTreg during the perioperative period. Data are presented as mean ± standard deviation. Comparisons were analyzed using two‐way repeated‐measures analysis of variance, followed by Bonferroni‐adjusted post hoc tests. T1, preoperative; T2, end of surgery; T3, 24 hours postoperatively; regulatory T, Treg; Interleukin‐10, IL‐10; transforming growth factor beta, TGF‐β. [file MCO2-7-e70840-s002.docx]

**Supplementary Material**

**Preoperative albumin infusion reduced pulmonary complications in elderly patients with hypoalbuminemia undergoing cardiac surgery: a single-center, randomized, double-blind, controlled pilot trial**

Jie Liu^1,2#^, Shiqiang Chen^2#^, Yunxiao Bai^2#^, Yong Lv^2^, Yanting Wang^2^, Zhenzhen Xu*^2^, Nianguo Dong^3*^, Qingping Wu^2*^

**Institutional affiliation(s):**

^1^ Department of Anesthesiology, The Second Affiliated Hospital, Chongqing Medical University, Chongqing, China.

^2^ Department of Anesthesiology, Union Hospital, Tongji Medical College, Huazhong University of Science and Technology, Wuhan 430022, China.

^3^ Department of Cardiovascular Surgery, Union Hospital, Tongji Medical College, Huazhong University of Science and Technology, Wuhan 430022, Hubei, China.

^#^ These three authors contributed equally to this work and share first authorship.

^*^ These three authors contributed equally to this work and share the corresponding author.

**Correspondence:**

Name: Zhenzhen Xu

Mailing address: Department of Anesthesiology, Union Hospital, Tongji Medical College, Huazhong University of Science and Technology, Wuhan 430022, China.

Email: 709675465@qq.com

Name: Nianguo Dong

Mailing Address: Department of Cardiovascular Surgery, Union Hospital, Tongji Medical College, Huazhong University of Science and Technology, Wuhan 430022, Hubei, China.

Email: [NG_Dong@126.com](mailto:NG_Dong@126.com)

Name: Qingping Wu

Mailing address: Department of Anesthesiology, Union Hospital, Tongji Medical College, Huazhong University of Science and Technology, Wuhan 430022, China.

Email: [wqp1968@163.com](mailto:wqp1968@163.com)

Table S1 Subgroup analyses of PPCs scores stratified by age, gender, preoperative albumin level, and surgical type.

| Subgroup | Control group  (n = 40) | Albumin group  (n = 40) | P value |
| --- | --- | --- | --- |
| Age, years |  |  |  |
| Age <70 | 3 (2–3) | 1 (1–2.25) | 0.037 |
| Age ≥70 | 3 (2–3) | 1 (1–3) | 0.01 |
| Gender |  |  |  |
| Female | 2 (2–3) | 1 (1–2) | 0.014 |
| Male | 3 (2–3) | 1 (1–3) | 0.018 |
| Preoperative Albumin level, g/L |  |  |  |
| Albumin <35 | 3 (2.25–3.75) | 1 (1–1.5) | 0.043 |
| 35 ≤Albumin<40 | 3 (2–3) | 1 (1–3) | 0.007 |
| Surgery type |  |  |  |
| CABG | 3 (2–3) | 1 (1–1.75) | 0.023 |
| Valve | 3 (2–3) | 2 (1–3) | 0.042 |
| Others | 2.5 (2–3) | 1 (1–3) | 0.106 |

Data are presented as median (interquartile range).

Abbreviations: PPCs, postoperative pulmonary complications.

Table S2 Baseline characteristics of patients with collected blood sampled.

| Variables | Control group  (n = 24) | Albumin group  (n = 24) | P value |
| --- | --- | --- | --- |
| Age, years | 68.5 (67, 72) | 68 (66, 71.25) | 0.395 |
| Male | 16 (66.7) | 16 (66.7) | 1 |
| BMI, kg/m^2^ | 23.83 ± 2.94 | 24.29 ± 3.66 | 0.635 |
| EuroSCORE | 4 (3,6) | 3.5 (2.25,3.5) | 0.309 |
| ARISCAT score | 50(50,50) | 50 (50,50) | 0.487 |
| NYHA | 1 (1,2) | 1.5 (1,1.5) | 0.566 |
| LVEF, % | 60 (58.75, 65) | 60 (56.55, 65.25) | 0.747 |
| SpO_2_, % | 98 (96.25,99) | 97 (96.35,97) | 0.534 |
| Smoking | 14 (58.3) | 14 (58.3) | 1 |
| Alcohol | 13 (54.2) | 12 (50) | 1 |
| Hypertension | 12 (50) | 15 (62.5) | 0.383 |
| Diabetes | 6 (25) | 4 (16.7) | 0.477 |
| Coronary heart disease | 15 (62.5) | 15 (62.5) | 1 |
| Atrial fibrillation | 5 (20.8) | 4 (16.7) | 1 |
| COPD | 1 (4.2) | 5 (20.8) | 0.19 |
| Stroke | 2 (8.3) | 3 (12.5) | 1 |
| Hemoglobin, g/L | 124.46 ± 12.95 | 124 ± 16.14 | 0.914 |
| ALT, U/L | 19 (16, 26) | 21 (16, 44) | 0.563 |
| AST, U/L | 22 (20, 33.25) | 24 (21, 33) | 0.62 |
| Albumin, g/L | 36.72 ± 1.94 | 36.98 ± 2.1 | 0.665 |
| Blood urea nitrogen, mmol/L | 6.55 ± 1.59 | 6.38 ± 1.88 | 0.73 |
| Creatinine, μmol/L | 76.81 ± 19.92 | 80.25 ± 20.33 | 0.557 |
| Type of surgery |  |  | 0.934 |
| CABG | 9 (37.5) | 9 (37.5) |  |
| Valve | 8 (33.3) | 9 (37.5) |  |
| Others | 7 (29.2) | 6 (25) |  |
| Intraoperative crystalloid, ml | 1500 (1075, 1500) | 1500 (1450, 2000) | 0.076 |
| Intraoperative colloid, ml | 0 (0,150) | 0 (0,0) | 0.274 |
| RBC transfusion, units | 1.25 (0,1.25) | 1.25 (0,1.25) | 0.472 |
| plasma, ml | 0 (0,0) | 0 (0,0) | 0.834 |
| Duration of surgery, min | 287.25 ± 67.42 | 266.33 ± 61.18 | 0.266 |
| Duration of anesthesia, min | 361.25 ± 85.09 | 327.88 ± 60.67 | 0.125 |
| CPB, min | 120.33 ± 36.98 | 117.29 ± 28.97 | 0.753 |
| aortic occlusion time, min | 75.17 ± 26.41 | 78.42 ± 23.53 | 0.655 |
| Pre-filled crystal, ml | 500（500,500） | 500（500,500） | 0.305 |
| Pre-filled with colloid, ml | 500 (500,725) | 700 (500,700) | 0.884 |
| Urine output, ml | 1625 (1437.5, 2225) | 1550 (1037.5, 1775) | 0.257 |

Data are presented as n (%) , mean (standard deviation), or median (interquartile range).

Abbreviations: BMI, body mass index; COPD, chronic obstructive pulmonary disease; NYHA, New York Heart Association; LVEF, left ventricular ejection fraction; SpO_2_, oxygen saturation; EuroSCORE, the European system for cardiac operative risk evaluation; ARISCAT, the assess respiratory risk in surgical patients in Catalonia; ALT, alanine aminotransferase; AST, aspartate aminotransferase; CABG, coronary artery bypass graft; CPB, cardiopulmonary bypass; RBC, red blood cells.

Table S2 Spearman correlation analysis between T cell subtypes and PPCs scores.

| T cell subtypes | Correlation Coefficient | P value |
| --- | --- | --- |
| Th1 difference (end of surgery minus baseline) | -0.026 | 0.863 |
| Th1 difference (24 hours after surgery minus baseline) | -0.133 | 0.366 |
| Th17 difference (end of surgery minus baseline) | 0.312 | 0.031 |
| Th17 difference (24 hours after surgery minus baseline) | 0.263 | 0.071 |
| Treg difference (end of surgery minus baseline) | -0.159 | 0.281 |
| Treg difference (24 hours after surgery minus baseline) | -0.017 | 0.911 |
| γδ1 difference (end of surgery minus baseline) | 0.02 | 0.895 |
| γδ1 difference (24 hours after surgery minus baseline) | 0.16 | 0.278 |
| γδ17 difference (end of surgery minus baseline) | -0.037 | 0.803 |
| γδ17 difference (24 hours after surgery minus baseline) | -0.142 | 0.341 |
| γδTreg difference (end of surgery minus baseline) | -0.302 | 0.037 |
| γδTreg difference (24 hours after surgery minus baseline) | -0.067 | 0.652 |

Abbreviations: PPCs, postoperative pulmonary complications. T helper, Th; regulatory T, Treg


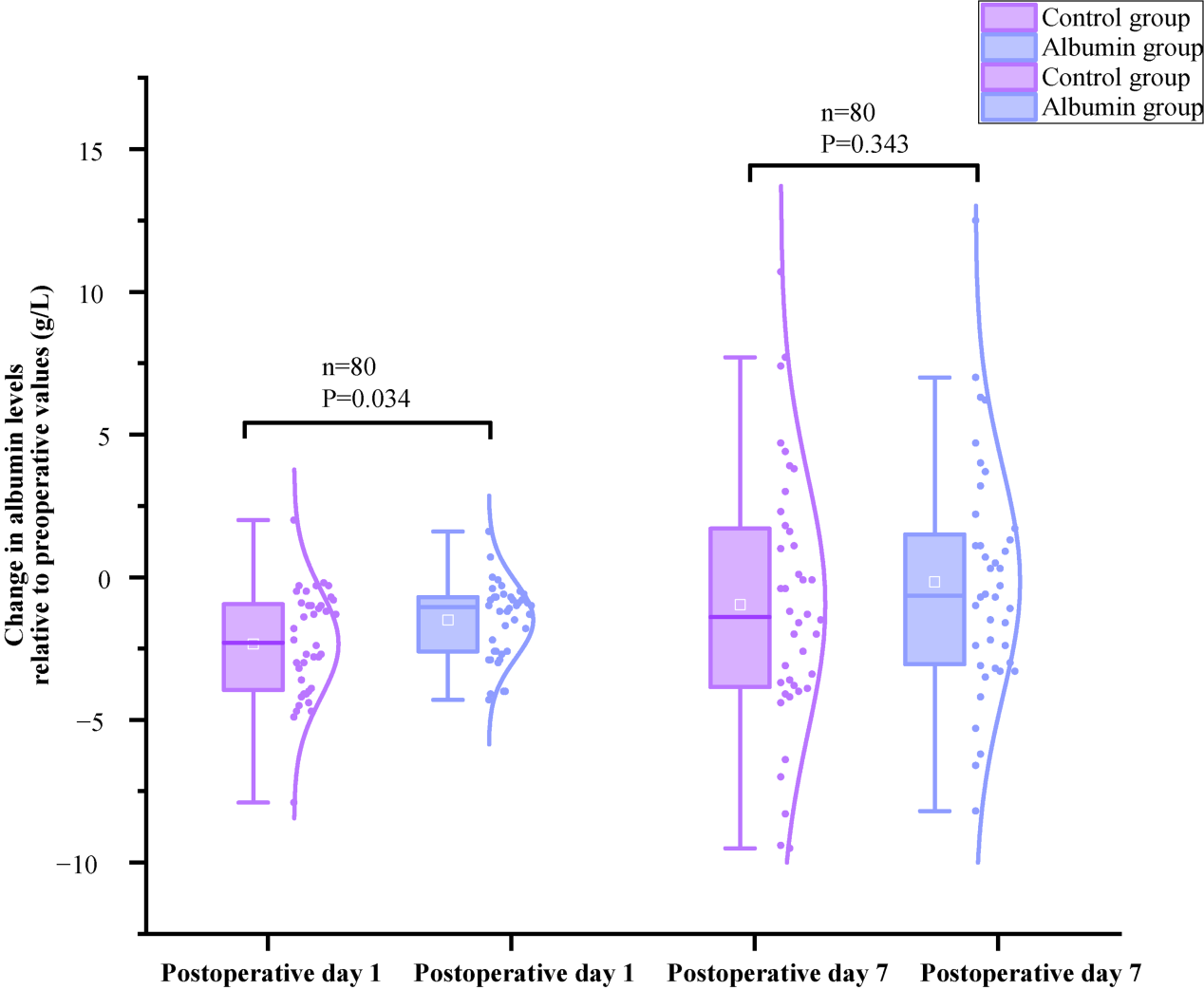


Figure S1. Changes in albumin concentration on postoperative day 1 and day 7 relative to preoperative levels.


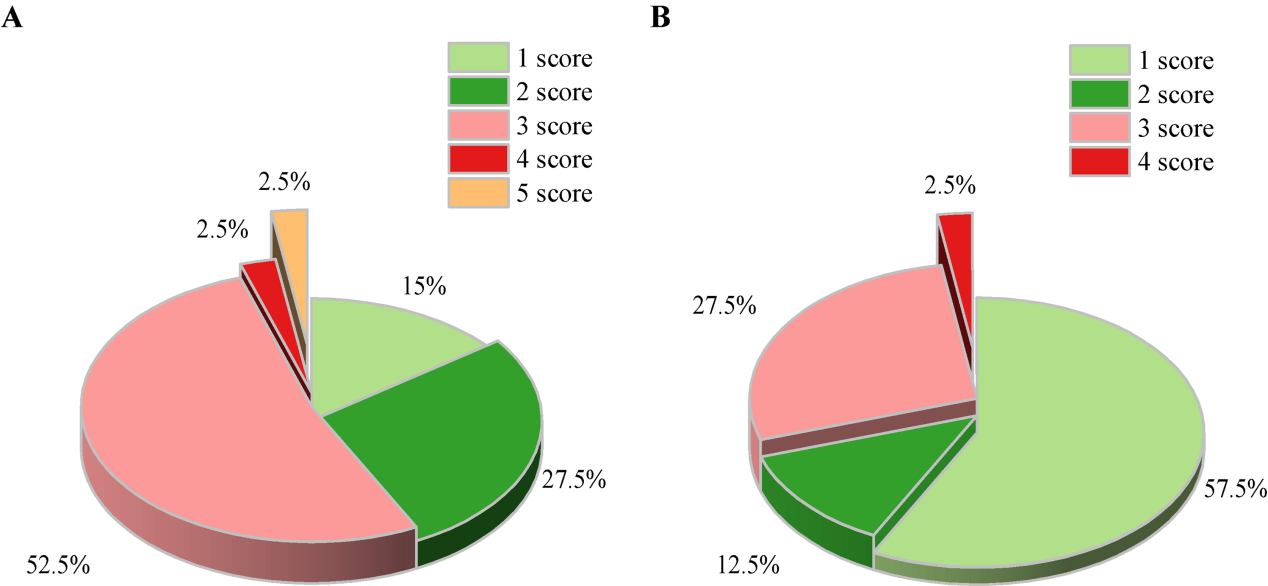


Figure S2. The PPCs score distribution in the albumin group and the control group. (A) The PPCs score distribution in the albumin group. (B) The scores of PPCs in the control group. PPCs, postoperative pulmonary complications.


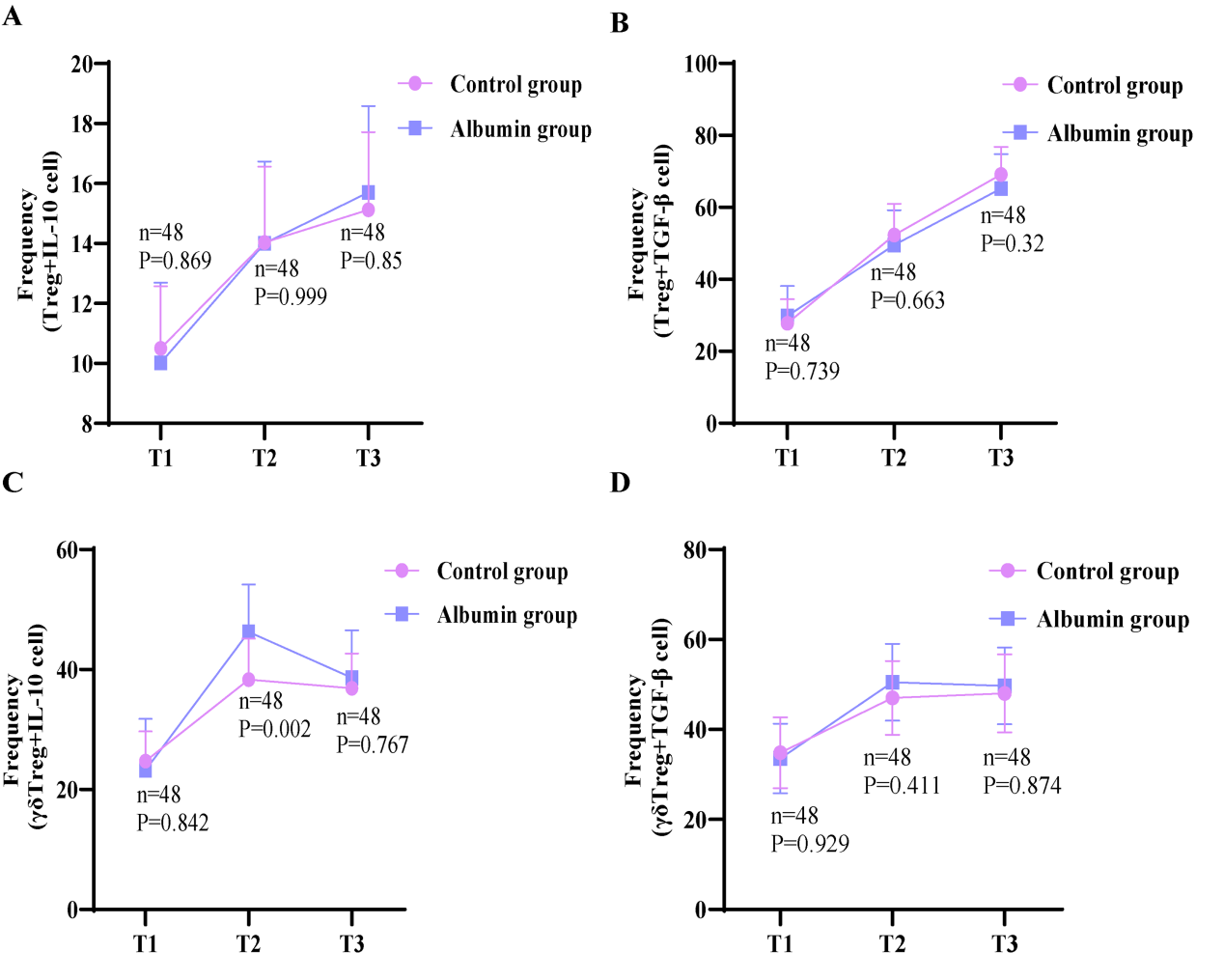


Figure S3. Changes in intracellular factors within perioperative Treg cells and γδTreg subsets. (A) Changes in IL-10 within Treg cells during the perioperative period. (B) Changes in TGF-β within Treg cells during the perioperative period. (C) Changes in IL-10 within γδTreg cells during the perioperative period. (D) Changes in TGF-β within γδTreg during the perioperative period. Data are presented as mean ± standard deviation. Comparisons were analyzed using two-way repeated-measures analysis of variance, followed by Bonferroni-adjusted post hoc tests. T1, preoperative; T2, end of surgery; T3, 24 hours postoperatively; regulatory T, Treg; Interleukin-10, IL-10; transforming growth factor beta,TGF-β.
