# Supplementary material for: Preoperative Albumin Infusion Reduced Pulmonary Complications in Elderly Patients With Hypoalbuminemia Undergoing Cardiac Surgery: A Single‐Center, Randomized, Double‐Blind, Controlled Pilot Trial
Source: MedComm (2020). 2026 Jul 5;7(7):e70840. doi: 10.1002/mco2.70840 (PMC13334133; doi:10.1002/mco2.70840)
Supplement: Supplementary file 2 — Supporting Information: mco270840‐sup‐0002‐SuppMat.docx [file MCO2-7-e70840-s001.docx]

**Supplemental Data -Study Protocol**
**Title**

Preoperative albumin infusion reduced pulmonary complications in elderly patients with hypoalbuminemia undergoing cardiac surgery: a single-center, randomized, double-blind, controlled pilot trial

**Background**

Postoperative pulmonary complications (PPCs) are a significant factor influencing postoperative outcomes, leading to increased morbidity and mortality, prolonged hospital length of stay, and are closely associated with adverse financial healthcare outcomes.^1,2^ Patients undergoing cardiac surgery are particularly susceptible to PPCs, with cardiopulmonary bypass (CPB) being a major contributing factor. CPB induces a systemic inflammatory response and oxidative stress, resulting in pulmonary ischemia-reperfusion injury. Additionally, mechanical ventilation is typically interrupted during CPB, further exacerbating the risk of atelectasis. It is estimated that approximately 1.25 million patients worldwide undergo CPB-assisted cardiac surgery annually.^3,4^ PPCs remain prevalent following CPB cardiac surgery, with severity ranging from mild hypoxemia to acute respiratory distress syndrome (ARDS).^5^ Therefore, optimizing perioperative lung protection strategies to reduce the incidence of PPCs remains a critical area of research in cardiac surgery anesthesia and postoperative management.

Albumin is one of the most abundant proteins in human blood and plays a crucial role in various physiological processes beyond its colloid osmotic function. Its primary functions include the transport of hydrophobic molecules, maintenance of endothelial glycocalyx integrity, and multiple biological activities such as antioxidant, anti-inflammatory, enzymatic, and signal regulatory functions.^6,7^ Studies have demonstrated that albumin can suppress inflammatory processes, thereby reducing microcirculatory disturbances and tissue damage.^8^ In addition to serving as a marker of nutritional status, albumin is also recognized as a biochemical indicator of inflammation.^9^

Multiple studies have identified hypoalbuminemia as an independent risk factor for PPCs,^10,11^ Our previous retrospective study involving 660 elderly patients undergoing cardiac surgery with CPB also revealed that a preoperative albumin level below 40 g/L was an independent risk factor for PPCs in this population. However, high-quality studies evaluating the impact of albumin infusion during cardiac surgery on PPCs remain limited.

This study aims to evaluate the clinical effects of exogenous albumin infusion compared to 0.9% saline. The primary objective is to assess the efficacy of albumin infusion in reducing the severity and incidence of PPCs. The secondary objectives include evaluating the duration of postoperative invasive mechanical ventilation, the incidence of postoperative acute kidney injury (AKI), the occurrence of postoperative cardiovascular complications, the length of ICU stay, the total length of hospital stay, all-cause postoperative mortality, and changes in T-cell subsets as measured by flow cytometry. By comprehensively analyzing pulmonary, renal, cardiovascular, and hospitalization-related outcomes, this study aims to systematically assess the clinical value of albumin infusion in perioperative management.

**Methods/Design**

**Design**

This study is a prospective, single-center, randomized controlled pilot trial designed to compare the clinical effects of two different perioperative management strategies in cardiac surgery with CPB. The intervention group receives an intravenous infusion of 100 mL of 20% human albumin (Kilifor, Spain, 50 mL: 10 g) immediately after venous access is established, whereas the control group receives 100 mL of 0.9% NaCl solution.

This study adopts a standardized infusion protocol of 100 mL of 20% human albumin (20 g) based on several key considerations. First, previous studies have demonstrated that hypoalbuminemia (<40 g/L) is strongly associated with an increased risk of postoperative pulmonary complications (PPCs). Therefore, this study includes patients with preoperative albumin levels ≥30 g/L, aiming to elevate plasma albumin levels to approximately 40 g/L. Given that each 10 g of 20% albumin can transiently increase plasma albumin concentration by 3–4 g/L, a 20 g infusion is expected to raise levels to the target range, thereby optimizing postoperative recovery. Second, standardizing the infusion to 20 g ensures consistency in intervention, preventing confounding factors associated with individualized dosing adjustments, thereby enhancing the reproducibility and scientific rigor of the study. Additionally, high-concentration (20%) albumin effectively increases plasma colloid osmotic pressure, reducing tissue fluid retention, postoperative pulmonary edema, and perioperative fluid overload, which is particularly beneficial for patients undergoing cardiopulmonary bypass (CPB) cardiac surgery. Finally, considering the high cost of albumin and the need to minimize waste, as well as to maintain the integrity of the study’s double-blind design, a uniform administration of two vials (20 g) was implemented. This approach ensures that all participants receive the same dose, thereby optimizing perioperative management and allowing for a robust evaluation of the impact of albumin infusion on postoperative complications.Therefore, this study adopts this dose regimen to ensure both safety and efficacy, aiming to optimize perioperative management and evaluate its impact on postoperative complications.

**Study population**

**Inclusion criteria**

Adult patients were consecutively enrolled based on the following criteria: age ≥65 years; undergoing primary cardiac surgery, either alone or in combination with CPB, including coronary artery bypass grafting (CABG), valve repair or replacement, and aortic surgery; preoperative serum albumin level between 30-40 g/L; signed informed consent.

**Exclusion criteria**

Exclusion criteria were as follows: history of allergy to albumin; preoperative use of high-dose positive inotropic agents, intra-aortic balloon pumps, extracorporeal membrane oxygenation (ECMO), or ventricular assist devices; preoperative renal insufficiency (serum creatinine ≥1.5 mg/dL) or dialysis dependency; left ventricular ejection fraction ≤40%; mechanical ventilation within 7 days prior to surgery; emergency procedures.

**Interventions**

The human albumin used in this study (manufactured by Grifols, Spain; 50 mL: 10 g) or the placebo (0.9% NaCl) was stored under sealed conditions to ensure stability and research integrity. To maintain the double-blind design and anonymity of drug allocation, all study medications were individually packaged in separate containers. Each participant’s assigned study medication was dispensed according to randomized enrollment numbers and was meticulously recorded in the case report form (CRF) to ensure data integrity and traceability.

The infusion of the study medication was administered intraoperatively, with the infusion initiated immediately after venous access was established. The medication—either human albumin or placebo—was delivered at a predefined rate, ensuring standardization and consistency in accordance with the study protocol.

**Standard procedures**

**Screening and inclusion**

During the preoperative visit, patients underwent screening based on the inclusion and exclusion criteria. If no exclusion criteria were met, patients were enrolled in the study after providing written informed consent.

**Randomization**

All patients who provided written informed consent were randomly assigned (1:1) to either the albumin group or the control group. Patient allocation was determined using a computer-generated randomization list, with the assignment sequence concealed from investigators to prevent selection bias.

**Blinding**

The trial was blinded for participants, meaning that patients were unaware of whether they were assigned to the albumin group or the control group. Additionally, blinding was applied to the responsible physicians, follow-up personnel, and statisticians. Upon the patient's transfer from the operating room, the ICU physicians remained unaware of the treatment allocation. Similarly, the research personnel responsible for follow-up and the data analysts had no knowledge of the patients’ group assignments, ensuring the integrity of the study.

**Procedure for unblinding if needed**

In the event of a serious adverse event requiring disclosure of the participant's group assignment, unblinding may be performed. The randomization list is securely maintained by an independent individual who is not involved in the study. In case of an emergency, investigators may contact the principal investigator, who is authorized to approve and coordinate access to the participant’s group assignment to ensure appropriate clinical management.

**Standard Care**

All patients enrolled in the study received a standardized anesthetic protocol to minimize the potential impact of anesthesia on pulmonary outcomes. Radial artery cannulation was performed under local anesthesia, followed by tracheal intubation and mechanical ventilation. Mechanical ventilation was performed using a lung-protective strategy with volume-controlled ventilation, a tidal volume of 6–8 mL/kg ideal body weight, and an inspiratory-to-expiratory ratio of 1:2. The respiratory rate was adjusted to maintain an end-tidal carbon dioxide level of 35–45 mmHg. The inspired oxygen fraction (FiO₂) was initially set at 100% during tracheal intubation, then reduced to approximately 60% after intubation, and subsequently titrated to maintain peripheral oxygen saturation (SpO₂ ≥96%) during surgery. Positive end-expiratory pressure of 3-5 cmH₂O was applied as part of routine clinical practice. During CPB, mechanical ventilation was discontinued according to institutional routine practice and resumed after weaning from CPB. All patients received a standardized ventilation protocol throughout the procedure.Anesthesia was induced and maintained using target-controlled infusion (TCI) of propofol and remifentanil, with non-depolarizing neuromuscular blocking agents administered to maintain skeletal muscle relaxation. The bispectral index (BIS) was maintained between 40 and 60 to ensure an appropriate depth of anesthesia. Intraoperatively, the infusion rates of vasoactive agents and fluids were adjusted as necessary based on the patient’s hemodynamic status.

At the conclusion of surgery, patients were transferred to the cardiac surgery intensive care unit (ICU) while still intubated for postoperative management. All patients were immediately transferred to the ICU for continuous monitoring and respiratory and circulatory support. Extubation was considered only when the following criteria were met: the patient exhibited stable respiratory and circulatory function, regained clear consciousness, demonstrated the ability to comprehend and follow simple commands, restored adequate muscle strength, maintained a respiratory rate greater than 10 breaths per minute, and sustained oxygen saturation comparable to preoperative levels after breathing room air for 5 minutes.

All other treatments were administered in accordance with standard clinical care protocols.

**Follow-up**

The follow-up period extends throughout the postoperative hospitalization, typically not exceeding 30 days.

**Blood Sample Collection and Flow Cytometry Analysis**

Arterial blood samples were collected from patients who provided informed consent for blood sampling and flow cytometry analysis at three time points: preoperatively, at the end of surgery, and 24 hours postoperatively. Blood sampling was performed via an arterial catheter, specifically before the infusion of albumin or saline, at the completion of surgery, and 24 hours postoperatively, to analyze the dynamics of Th1, Th17, Treg, γδ1, γδ17, and γδTreg cell populations.

During the experimental process, whole blood samples underwent erythrocyte lysis to remove red blood cells, thereby optimizing leukocyte isolation efficiency and minimizing interference from non-target cells. The isolated leukocytes were then subjected to sequential processing, including cell stimulation, viability staining, surface staining, and intracellular staining, ensuring the precise identification of different T cell subsets. Following staining, the cells were washed twice with PBS to remove unbound antibodies and dyes, and subsequently resuspended in PBS to preserve cell viability and signal stability. Data acquisition was performed using a FACS Fortessa flow cytometer (BD, San Diego, CA, USA) to ensure high-throughput detection accuracy and reproducibility.

The study utilized a comprehensive panel of antibodies targeting T cell subsets and cytokines, including APC-H7-CD3, BV605-CD4, BV421-TCRγδ, APC-IL-17A, BV510-IFN-γ, PE-Foxp3, PE-CY7-TGF-β1, BB700-IL-10, and isotype-matched control IgG. All antibodies were purchased from BD PharMingen or BioLegend (San Diego, CA, USA). The selection of antibodies was based on validated literature evidence to ensure specificity and sensitivity of the experimental results.

Flow cytometry data were analyzed using FlowJo software (BD, San Diego, CA, USA), employing a standardized gating strategy to ensure data accuracy, consistency, and reproducibility. To minimize potential bias during analysis, all flow cytometry data underwent independent quality control by at least two researchers, thereby enhancing scientific rigor and reliability.

**Study Endpoints**

The primary endpoints of this study are the severity and incidence of PPCs during hospitalization. The definition of PPCs is based on the scoring system proposed by Kroenke et al.,^12^ as detailed below.

Grade 1:

-Cough, dry

-Microatelectasis: abnormal lung findings and temperature > 37.5°C without other documented cause; normal chest radiograph

-Dyspnea, not due to other documented cause

Grade 2: (We only classified as grade 2 if two or more items in the grade 2 were present.)

-Cough, productive, not due to other documented cause

-Bronchospasm: new wheezing or pre-existent wheezing resulting in a change in therapy

-Hypoxemia (SpO₂ ≤ 90%) at room air

-Atelectasis: gross radiological confirmation (concordance of 2 independent experts) plus either temperature > 37.5°C or abnormal lung findings

-Hypercarbia (PaCO₂ > 50 mmHg), requiring treatment

Grade 3:

-Pleural effusion, resulting in thoracentesis

-Pneumonia: radiological evidence (concordance of 2 independent experts) plus clinical symptoms (two of the following: leucocytosis or leucopenia, abnormal temperature, purulent secretions), plus either a pathological organism (by Gram stain or culture), or a required change in antibiotics

-Pneumothorax

-Noninvasive ventilation, strictly applied to those with all of the following: Oxygen saturation (SpO₂) lower than 92% under supplemental oxygen; Need of supplemental oxygen > 5 L/min; Respiratory rate (RR) ≥ 30 bpm.

-Re-intubation postoperative or intubation, period of ventilator dependence (non-invasive or invasive ventilation) ≤ 48 hours

Grade 4: Ventilatory failure: postoperative ventilator dependence exceeding 48 hours, or reintubation with subsequent period of ventilator dependence exceeding 48 hours

Grade 5: Death before hospital discharge。

A PPC was defined as a PPCs score ≥3, with both the incidence and specific types of complications recorded accordingly. All patients underwent bedside chest radiographs on postoperative days 1, 3, and 5, followed by weekly imaging during hospitalization, unless otherwise indicated by the attending physician. All chest radiographs and follow-up assessments were independently analyzed by two trained researchers who were blinded to the study group assignments. The final PPCs score was determined only after ensuring consistency between radiographic findings and clinical manifestations. The scoring system ranges from a minimum of 0 to a maximum of 5.

Secondary endpoints include the duration of postoperative invasive mechanical ventilation, incidence of postoperative acute kidney injury (AKI), occurrence of postoperative cardiovascular complications, length of ICU stay, total hospital length of stay, and all-cause postoperative mortality.

**Sample size**

Based on the published literature,^13,14^ and the results of the pre-test, we estimated that the difference in postoperative pulmonary complications scores between the intervention and control groups would be 0.3, with a standard deviation of 0.45. For postoperative pulmonary complications incidence, we assumed a rate of 60% in the control group and anticipated a 50% relative reduction in the intervention group. Using a two-sided test with a significance level (α) of 0.05 and a power of 80%, the required sample size was calculated separately for each outcome. The sample size estimation based on postoperative pulmonary complications scores resulted in a requirement of 74 patients, whereas the calculation based on postoperative pulmonary complications incidence indicated a requirement of 58 patients. To ensure sufficient statistical power, the larger sample size of 74 was adopted. Accounting for a dropout rate of 5%, the final recruitment target was set at 80 patients.

**Statistical Analysis**

The baseline characteristics of patients will be summarized and analyzed using descriptive statistical methods. Normality will be assessed using the Kolmogorov-Smirnov test or the Shapiro-Wilk test. Normally distributed continuous variables will be presented as mean ± standard deviation (mean ± SD), whereas non-normally distributed continuous variables will be expressed as median and interquartile range (median [IQR]). Categorical variables will be reported as frequency and percentage (n, %). Group comparisons will be conducted using appropriate statistical methods based on the type of variable. Independent samples t-tests will be used for normally distributed continuous variables, while the Mann-Whitney U test will be applied for non-normally distributed continuous variables. Categorical variables will be compared using the chi-square test or Fisher’s exact test (for expected counts <5). Subgroup analyses were performed according to preoperative subgroup analyses for the PPC severity score, the primary continuous outcome of the study. Patients were stratified according to preoperative serum albumin levels (<35 g/L and 35–40 g/L) on the basis of the clinically relevant thresholds for hypoalbuminemia. Additional age, sex, and surgical type subgroup analyses were performed to evaluate the consistency of the treatment effect across different patient characteristics and procedures. A two-sided P value of less than 0.05 was considered to indicate statistical significance. All the statistical analyses and visualizations were performed using SPSS software version 25.0 (IBM, Armonk, NY, USA), Origin (OriginLab Corporation, Northampton, MA, USA), and GraphPad Prism (GraphPad Software, San Diego, CA, USA).

For flow cytometry measurements at different time points, Mauchly’s test will be conducted to assess the assumption of sphericity. If the assumption is met, repeated-measures analysis of variance (repeated-measures ANOVA) will be used, followed by Bonferroni post hoc correction for multiple comparisons. If the assumption is violated, Greenhouse-Geisser correction or the Friedman test (for non-normally distributed data) will be applied. Additionally, the correlation between changes in T-cell subset counts and PPC scores will be analyzed using Spearman’s rank correlation test; Pearson’s correlation analysis will be used if the data are normally distributed. All statistical analyses will be conducted using two-sided tests, with a P-value <0.05 considered statistically significant. For multiple comparisons, P-values will be adjusted using Bonferroni correction. Data processing and visualization will be performed using SPSS 26.0 (IBM, Armonk, NY, USA), Origin (OriginLab Corporation, Northampton, MA, USA), GraphPad Prism (GraphPad Software, San Diego, CA, USA), and R 4.4.0.

**References**

1. Fernandez-Bustamante, A., Frendl, G., Sprung, J., Kor, D.J., Subramaniam, B., Martinez Ruiz, R., Lee, J.W., Henderson, W.G., Moss, A., Mehdiratta, N., et al. (2017). Postoperative Pulmonary Complications, Early Mortality, and Hospital Stay Following Noncardiothoracic Surgery: A Multicenter Study by the Perioperative Research Network Investigators. JAMA Surg *152*, 157-166. 10.1001/jamasurg.2016.4065.

2. Stéphan, F., Barrucand, B., Petit, P., Rézaiguia-Delclaux, S., Médard, A., Delannoy, B., Cosserant, B., Flicoteaux, G., Imbert, A., Pilorge, C., and Bérard, L. (2015). High-Flow Nasal Oxygen vs Noninvasive Positive Airway Pressure in Hypoxemic Patients After Cardiothoracic Surgery: A Randomized Clinical Trial. Jama *313*, 2331-2339. 10.1001/jama.2015.5213.

3. Colomina, N., Garí, E., Gallego, C., Herrero, E., and Aldea, M. (1999). G1 cyclins block the Ime1 pathway to make mitosis and meiosis incompatible in budding yeast. Embo j *18*, 320-329. 10.1093/emboj/18.2.320.

4. Lagier, D., Fischer, F., Fornier, W., Huynh, T.M., Cholley, B., Guinard, B., Heger, B., Quintana, G., Villacorta, J., Gaillat, F., et al. (2019). Effect of open-lung vs conventional perioperative ventilation strategies on postoperative pulmonary complications after on-pump cardiac surgery: the PROVECS randomized clinical trial. Intensive Care Med *45*, 1401-1412. 10.1007/s00134-019-05741-8.

5. Ng, C.S., Wan, S., Yim, A.P., and Arifi, A.A. (2002). Pulmonary dysfunction after cardiac surgery. Chest *121*, 1269-1277. 10.1378/chest.121.4.1269.

6. Zeng, Y., Adamson, R.H., Curry, F.R., and Tarbell, J.M. (2014). Sphingosine-1-phosphate protects endothelial glycocalyx by inhibiting syndecan-1 shedding. Am J Physiol Heart Circ Physiol *306*, H363-372. 10.1152/ajpheart.00687.2013.

7. Ferrer, R., Mateu, X., Maseda, E., Yébenes, J.C., Aldecoa, C., De Haro, C., Ruiz-Rodriguez, J.C., and Garnacho-Montero, J. (2018). Non-oncotic properties of albumin. A multidisciplinary vision about the implications for critically ill patients. Expert Rev Clin Pharmacol *11*, 125-137. 10.1080/17512433.2018.1412827.

8. Hariri, G., Joffre, J., Deryckere, S., Bigé, N., Dumas, G., Baudel, J.L., Maury, E., Guidet, B., and Ait-Oufella, H. (2018). Albumin infusion improves endothelial function in septic shock patients: a pilot study. Intensive Care Med *44*, 669-671. 10.1007/s00134-018-5075-2.

9. Shen, S., and Xiao, Y. (2023). Association Between C-Reactive Protein and Albumin Ratios and Risk of Mortality in Patients with Chronic Obstructive Pulmonary Disease. Int J Chron Obstruct Pulmon Dis *18*, 2289-2303. 10.2147/copd.S413912.

10. Hu, Y., Wang, L., Liu, H., Yang, K., Wang, S., Zhang, X., Qu, B., and Yang, H. (2023). Association of preoperative hypoprotein malnutrition with spinal postoperative complications and other conditions: A systematic review and meta-analysis. Clin Nutr ESPEN *57*, 448-458. 10.1016/j.clnesp.2023.07.083.

11. Chen, Y., Wu, G., Wang, R., and Chen, J. (2018). Preoperative Albumin Level Serves as a Predictor for Postoperative Pulmonary Complications Following Elective Laparoscopic Gastrectomy. Curr Pharm Des *24*, 3250-3255. 10.2174/1381612824666180713104307.

12. Kroenke, K., Lawrence, V.A., Theroux, J.F., and Tuley, M.R. (1992). Operative risk in patients with severe obstructive pulmonary disease. Arch Intern Med *152*, 967-971.

13. Costa Leme, A., Hajjar, L.A., Volpe, M.S., Fukushima, J.T., De Santis Santiago, R.R., Osawa, E.A., Pinheiro de Almeida, J., Gerent, A.M., Franco, R.A., Zanetti Feltrim, M.I., et al. (2017). Effect of Intensive vs Moderate Alveolar Recruitment Strategies Added to Lung-Protective Ventilation on Postoperative Pulmonary Complications: A Randomized Clinical Trial. Jama *317*, 1422-1432. 10.1001/jama.2017.2297.

14. Wang, D., Wang, M., Zhang, H., Zhu, H., Zhang, N., and Liu, J. (2020). Effect of Intravenous Injection of Vitamin C on Postoperative Pulmonary Complications in Patients Undergoing Cardiac Surgery: A Double-Blind, Randomized Trial. Drug Des Devel Ther *14*, 3263-3270. 10.2147/dddt.S254150.
